# Micro- and Nanoplastics’ Effects on Protein Folding and Amyloidosis

**DOI:** 10.3390/ijms231810329

**Published:** 2022-09-07

**Authors:** Joseph Windheim, Laura Colombo, Nora C. Battajni, Luca Russo, Alfredo Cagnotto, Luisa Diomede, Paolo Bigini, Elena Vismara, Ferdinando Fiumara, Silvia Gabbrielli, Alfonso Gautieri, Gemma Mazzuoli-Weber, Mario Salmona, Luca Colnaghi

**Affiliations:** 1 Department of Medicine, NYU Grossman School of Medicine, New York, NY 10016, USA; 2 Department of Molecular Biochemistry and Pharmacology, Istituto di Ricerche Farmacologiche Mario Negri IRCCS, 20156 Milan, Italy; 3 Department of Chemistry, Materials and Chemical Engineering “G. Natta”, Politecnico di Milano, 20156 Milan, Italy; 4 Rita Levi Montalcini Department of Neuroscience, University of Torino, Corso Raffaello 30, 10125 Torino, Italy; 5 National Institute of Neuroscience (INN), University of Torino, Corso Raffaello 30, 10125 Torino, Italy; 6 Biomolecular Engineering Lab, Dipartimento di Elettronica, Informazione e Bioingegneria, Politecnico di Milano, Piazza Leonardo da Vinci 32, 20133 Milano, Italy; 7 Center for Systems Neuroscience (ZSN), 30559 Hannover, Germany; 8 Institute for Physiology and Cell Biology, University of Veterinary Medicine Hannover, 30559 Hannover, Germany

**Keywords:** plastics, micro- and nanoplastics, amyloids, Alzheimer’s disease, protein aggregation

## Abstract

A significant portion of the world’s plastic is not properly disposed of and, through various processes, is degraded into microscopic particles termed micro- and nanoplastics. Marine and terrestrial faunae, including humans, inevitably get in contact and may inhale and ingest these microscopic plastics which can deposit throughout the body, potentially altering cellular and molecular functions in the nervous and other systems. For instance, at the cellular level, studies in animal models have shown that plastic particles can cross the blood–brain barrier and interact with neurons, and thus affect cognition. At the molecular level, plastics may specifically influence the folding of proteins, induce the formation of aberrant amyloid proteins, and therefore potentially trigger the development of systemic and local amyloidosis. In this review, we discuss the general issue of plastic micro- and nanoparticle generation, with a focus on their effects on protein folding, misfolding, and their possible clinical implications.

## 1. Introduction

In 1970, the explorer Thor Heyerdahl captained an expedition across the Atlantic Ocean, from Morocco to Barbados. Over the course of his journey, he documented the pollution he encountered, including abundant plastic waste [1]. This was, perhaps, the first detailed account of oceanic pollution. He presented his findings to the United Nations, and on that basis, the United States Congress passed the Ocean Dumping Act in 1972, regulating waste dumping activities [1]. However, 50 years later, plastic waste has not diminished; on the contrary, it has risen to dramatic levels. The last 70 years have witnessed a tremendous increase in the global manufacturing of plastic products, reaching a production of nearly 360 million tons in 2018 (Plastics Europe, 2019), up from 2 million tons in 1950 [2]. That figure is expected to increase several-fold in the coming years, especially with the ever-increasing popularity of “single-use” plastic products (e.g., food and beverage packaging), whose use has further increased in response to the COVID-19 pandemic (i.e., masks, gloves, and food-related containers) [3]. In parallel to the increased plastic production, the amount of plastic waste is obviously growing and, due to its slow rate of degradation, the total amount of plastic waste will continue to accumulate in the environment, reaching a projected amount of 12 billion tons by 2050 [2,4]. The ‘mountains’ of discarded products and packaging that populate landfills [5] and accumulate in the oceans (e.g., the ‘great Pacific garbage patch’ [6]), made up of items ranging from synthetic teabags [7] to automobile tires [8], are alarming reminders of the ubiquity of plastics use in our societies. All this plastic material will eventually degrade into smaller particles which, if not properly managed, will end up in terrestrial and marine environments. Indeed, runoff from land-based sources, particularly coastal urban centers, accounts for 80% of the plastic load in marine environments [9]. More recent research has revealed that plastic debris is also commonly found in soil, although data on terrestrial deposits are relatively sparse [10].

During the degradation process, plastics release smaller fragments, called micro- or nanoplastics (MNPs) [11]. Microplastics (MPs) are defined as plastic particles <5 mm in diameter [12]. After additional erosion, MPs become nanoplastics (NPs), particles with at least one dimension <100 nm (EU Commission, 2011) recommendation of 18 October 2011 on the definition of nanomaterial (2011/696/EU). Collectively, MNPs are also referred to as “plastic debris” in the literature. It is worth noting that alternative size classifications have been proposed, though are not as widely accepted [13]; for instance, some consider MPs those particles <10 mm in diameter [14]. MNPs are not only generated after plastic items have been disposed of. Indeed, they can also be generated from the most routine everyday activities, such as cutting open plastic packaging [15] or through “wear and tear” of plastic tools. Moreover, in some cases, MPs are intentionally manufactured as microscopic pellets. These pellets are used in a variety of commercial products such as, for instance, hand and face scrubs which can contain exfoliating polyethylene “microbeads” [9,16,17]. Similarly, MPs are used as industrial abrasives which, if not properly controlled and disposed of, leach into the environment surrounding the factories [9,18]. MNPs are also added to medications as vectors for pharmaceutical agents [19]. However, the same adsorbent properties of MPs that make them attractive as pharmacological vectors also make them effective vehicles for the entry of pollutants and heavy metals into the human food chain [20,21].

The most abundant plastic polymers, accounting for 90% of those produced, are low-density polyethylene (LDPE), high-density polyethylene (HDPE), polyvinyl chloride (PVC), polystyrene (PS), polypropylene (PP), and polyethylene terephthalate (PET) [22]. A comprehensive list of the types of plastic and their most common applications has been compiled by Li et al. [9]. In general, the degradation of plastic polymers usually occurs via thermal reactions, photo-oxidation, microorganism breakdown, and mechanical disintegration [23,24]. Each polymer has specific physico-chemical properties (e.g., water solubility) that define its specific degradation behavior. An exemplary study of plastic degradation in the environment by Lambert et al. demonstrated that it takes only 14 days of immersion in water for a piece of a disposable polystyrene cup to begin producing MPs [25]. During the degradation process, plastics can also interact with the environment, which can modify their chemical properties, such as the net electrical charge, thus ultimately affecting their behavior and interactions with organic molecules [9,26].

## 2. Absorption of Plastics by Organisms

Toxicology studies carried out in the early 1990s revealed that polystyrene microspheres ranging from 50 nm to 3 μm in size (mimicking, therefore, both MPs and NPs), when administered by gavage to female Sprague Dawley rats, could be absorbed across the gastrointestinal tract and reach the lymph nodes, the liver, and spleen [27]. The gastrointestinal absorption was dependent on the quantity and the size of the particles. Additional studies demonstrated that the charge of the plastics may also influence the absorption [28]. More recent work has found that gastrointestinal absorption of MNPs also occurs in wild animals [29]. Plastic debris, ubiquitous in both marine and terrestrial habitats, is indeed inevitably consumed by those animals at the bottom of the food chain. Both zooplankton and C. elegans, the most studied primary consumers in their respective environments, feed relatively indiscriminately; this results in inadvertent consumption of MNPs. In zooplankton, the consumed particles concentrate in the mid-gut, where they can remain for up to one week [30]. The prolonged presence of particles within the organisms’ digestive tract facilitates the transmission of plastic debris through the trophic system, with bioamplification occurring at each step. Plastic particles and fibers have been identified in a multitude of species [31] that take them up directly from the water, soil, and air, or indirectly via the consumption of prey, and marine animals, which are particularly susceptible to plastic ingestion [32]. Humans are not spared, with the consumption of MPs evidenced by their presence in human stool [33]. The average American consumes an estimated 74,000 to 121,000 particles annually, a large portion of which comes in the form of shellfish, seawater-derived table salt, bottled water, and other beverages, industrial products (such as toothpaste), and via inhalation of MPs released from fabrics, rubber tires, and brakes [8,34,35,36].

These exposures result in the accumulation of MPs in different organs [37]. MPs have been detected in human lungs [34], blood [38], and placenta [39]. Nevertheless, whether or not exposure of humans to MNPs might have any health effects is still unclear [40]. In this sense, it is of extreme importance, starting from evidence in lower organisms [41], to study the mechanisms used by the NPs to cross the intestinal barrier and to take into account that this could be increased in case of gastrointestinal disease with inflammation or compromised epithelial functions (e.g., celiac disease, food intolerance, intestinal bowel disease).

## 3. In Vivo Toxicity and Neurotoxicity of MNPs

Experiments in model organisms, including mammals, have shown that ingested plastic particles can spread throughout the organism’s body, with deleterious effects on numerous organ systems, at the gross, histologic, and metabolic levels [42,43]. Yung-Li Wang et al. recently compiled a list of targeted organs, cataloged by organism [44]. The toxic effects in several species reported for ingested/inhaled plastic particles have been reported for most organs and are diverse. The particles can be accumulated into tissues, especially in the liver and intestines, causing toxicity, dysfunction, inflammation, alteration of gene expression profiles, increase oxidative lesions, and metabolic changes [44]. They also promote immune dysregulation through the disruption of circulating neutrophils’ degranulation capacity [45,46]. In fish, the particles may be able to deposit in the lipid-rich brain tissue, causing behavioral changes and lowering organismal fitness [47,48,49,50,51]. Experiments in worms and fish have shown that exposure to NPs correlates with increased expression of numerous genes involved in essential functions, particularly oxidation-reduction processes [52,53,54]. In neurons of invertebrates, NPs appear to upregulate neurotransmitter precursors [55] and downregulate acetylcholine (ACh) and gamma-aminobutyric acid (GABA) reuptake transporters [54], both mechanisms of which are indications of neurotoxicity. All these dysregulation processes are supposed to affect the organisms’ behaviors. Studies show that following NP ingestion, zebrafish larvae and adult fish became hypoactive, swim slower, and feed less efficiently [56,57]. Histological examinations revealed that the zebrafish brains appeared significantly more edematous, a reflection of either the NPs’ direct cytotoxicity or their osmotic effect once inside the neurons [56,57]. However, there is less evidence available to explain the effects of MNPs on mammalian brain alterations and physiology. It is still unclear whether MNPs could be detected in the cerebral tissues of mammals, humans included. In one of the few articles describing the neurobehavioral effects of long-term exposure to NPs in rats, the authors did not find major effects of polystyrene NPs [58]. However, they found subtle and transient behavioral effects in all groups of treated rats in comparison to the untreated ones. This indicates the urge for additional studies, on larger populations, to shed more light on these effects [43,59,60].

## 4. In Vitro MNP Effects

In vitro experiments have been performed to understand how NPs can affect cellular processes. First, it was shown that NPs can cross biological plasma membranes. Studies carried out in immortalized cells have shown that NPs with different sizes (40 nm vs. 150 nm) can be internalized via different mechanisms such as passive diffusion [61,62], clathrin-mediated, and caveolin-mediated endocytosis pathways, and micropinocytosis [62], depending on particle size. Once inside the cells, plastic particles appear to have significant effects on genome maintenance and gene regulation. For example, 100 nm polystyrene NPs induce reactive oxygen species formation and micronuclei, suggesting the onset of extensive DNA lesions upon exposure [63]. Polystyrene MPs (10 µm) increase the amount of active oxygen (ROS) in T98G cells, a cell line derived from a human glioblastoma multiform tumor [64]. Microglia, the central nervous system glial cells with immune functions, can absorb carboxylated polystyrene NPs through phagocytosis, suggesting the potential for neuroglia inflammation [65]. Cultured human dopaminergic neurons and neurospheres can take up polyethylene NPs (33 nm), causing changes in gene expression and an increase in malondialdehyde (MDA) levels, thereby indicating the emergence of oxidative stress [66,67].

## 5. MNPs and Proteins

The interaction of nanoparticles with proteins is the basis of nanoparticle bioreactivity [68]. Protein coatings may affect cell insertion, inflammation, accumulation, fission, and nanoparticle clearance. Alternatively, the surface of the nanoparticles can cause changes corresponding to the adsorbed protein molecules, which may affect the overall bioreactivity of the nanoparticles [69]. This is true for MPs and NPs as well. NPs, especially, are two orders of magnitude smaller than eukaryote cells, and therefore, they can be internalized by cells [70] and alter cellular structures (e.g., cell membrane) at the molecular level [71]. Once internalized, NPs can interact with biomolecules in the cytoplasm. Interaction of NPs with proteins can lead to three consequences: (i) protein corona formation, (ii) protein-induced coalescence of NPs, and (iii) conformational changes of protein secondary structure [72]. Although the binding mechanism between proteins and NPs is not completely clear, recent findings suggest that proteins interact with NPs mainly via weak interactions, such as hydrophobic interactions, hydrogen bonds, Van der Waals attraction forces, and electrostatic forces [73]. The formation of the protein corona depends on several parameters such as the size, shape, and chemical composition of the NPs, but also the medium (i.e., type of proteins and other chemical species), the duration of exposure, and the NPs/protein ratio. A molecular dynamic study [74] revealed that the interactions between insulin and polystyrene NPs are primarily driven by Van der Waals forces and hydrophobic interactions: these interactions occur primarily due to apolar amino acids such as leucine and alanine. NPs immersed in biological fluids combine with proteins in a dynamic process, and their interaction produces NP-protein complexes. Proteins can surround NPs to form a layer named protein corona. The adsorption of the proteins on the NP-surface provides them a new biological identity: coronated-NPs can escape from the immune system and interfere with cellular and molecular processes [72].This protein-envelopment is composed of an inner layer, called the hard corona, constituted by proteins that have a high binding affinity with NP surfaces, and an external layer, or soft corona, composed of proteins that show a lower binding affinity. The proteins in the soft corona are indirectly associated with the NP due to their interactions with the proteins of the hard corona, and they can easily interact with other proteins present in the environment. The hard- and soft-corona dictate the biological effects of the NPs since the protein corona is what is sensed by the immune systems, driving its response to the presence of the NPs. The presence of a protein corona also allows NPs to interact with each other through protein–protein interactions, promoting coalescence and aggregation [75].

## 6. MNP Effects on Protein Secondary Structures

The function of proteins is closely related to their three-dimensional structure: changes in the structure can cause protein misfolding and loss of functions. Evidence of secondary structure modifications and consequent misfolding in the presence of NPs such as polyethylene and polystyrene have been discussed by Hollóczki and colleagues [76] using molecular dynamic simulations. NP-protein interactions are evaluated as a function of the type of amino acids: apolar side chains, such as phenylalanine and tryptophan, are prone to adsorption on the surface of NP due to hydrophobic interactions. Hollóczki and coworkers also studied the interaction between various NPs and small peptides, predominantly characterized by different secondary structures (e.g., α-helices or β-sheets). A series of molecular dynamics simulations demonstrated that interactions with polyethylene NPs increase the presence of α-helices, while interaction with nylon NPs causes the unfolding of the helical domains while promoting a β-sheet-like structure. These results suggest that NPs may cause protein misfolding [76]. Further computational studies provide additional evidence of protein denaturation and conformational changes in the presence of NPs: when peptide models characterized by different secondary structures were simulated in the presence of polyethylene and nylon NPs, both NPs were found to influence the stability of the native secondary structures of the peptides [74,76,77].

## 7. MNPs and Aberrant Protein Folding 

**Figure 1:**
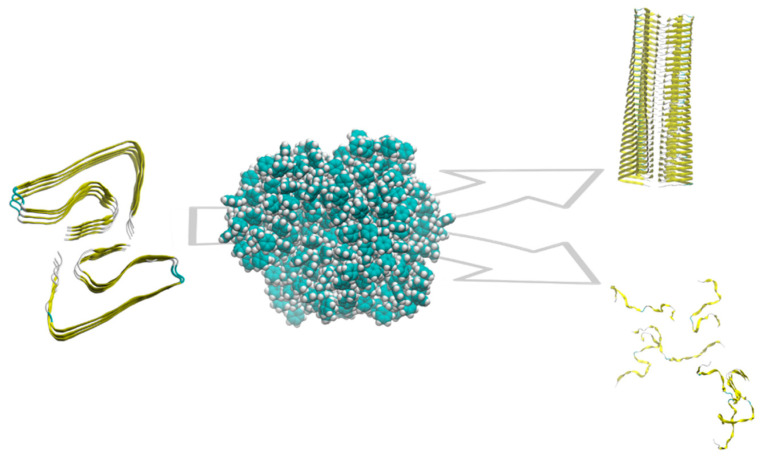
Schematic of the role of polystyrene NP on amyloid aggregation. Depending on the features of the NP and the protein/NP ratio, the aggregation is promoted or inhibited.

Protein fibrillation is defined as a dynamic process by which misfolded proteins form large oligomeric aggregates or amyloid fibrils. This mechanism of protein aggregation is involved in many human diseases, including Parkinson’s (PD) and Alzheimer’s diseases (AD). Many proteins and peptides can interact to form amyloid fibrils, including amyloid-beta peptides (i.e.,: 1-40 and 1-42), the prion protein, α-synuclein, tau, and β2-microglobulin. In most cases, protein or peptide aggregation is associated with increasing content in beta structures. Fibril formation begins with a slow interaction between misfolded proteins and preformed oligomers, which act as nuclei for the elongation of fibrils. The kinetics of protein fibrillation consists of three phases: the lag (nucleation) phase, the elongation phase, and the saturation phase. The process typically displays a sigmoidal growth curve. In particular, the nucleation phase is assumed to be the activation time required for the formation of “seeds”, from which fibrillation starts. NPs, such as polymer particles, are found to influence the rate of protein fibrillation [78]. Linse et al. [79] demonstrated in vitro that NPs enhance the probability of appearance of seeds for nucleation of fibrils, using human β2-microglobulin. They observed a shorter lag phase in presence of NPs and a connection with the amount of particle surface. These results show a “nanoplastic-assisted” mechanism, in which NPs act as seeds for nucleation. Amyloid β peptides (Aβ) are the predominant peptides found in the brain of patients with AD. Deposition of Aβ is an early event in the pathogenesis of AD: a strong correlation is reported to exist between the extent of free radical generation by Aβ and neurotoxicity. In addition to its direct neurotoxic effects, Aβ may also fragment into free radical peptides, more cytotoxic oligomers containing 25–35 amino acids [48]. The process leading to the formation of amyloid fibrils and plaques is called fibrillogenesis: insulin can form amyloid fibrils and is typically selected as a model protein to study the fibrillation process. Li et al. [74] examined the fibrillation of insulin promoted by polystyrene NPs. The results suggest that the presence of NPs decreased the lag phase time for insulin fibrillation, but there is no significant effect on the other two phases. Contrarily, Cabaleiro-Lago et al. [80] have shown that the presence of copolymeric NPs leads to a significant increase in the lag phase time of Aβ fibrillation, but does not completely stop fibril formation: elongation and saturation phases are unaffected by the presence of the NPs. Another study [81] showed an inhibitory effect of polymeric NPs on the fibrillation process, using PEGylated phospholipid nanomicelles. A following study by Cabaleiro-Lago et al. [82] investigated the effect of NPs on the fibrillation kinetics of the Aβ peptide using polystyrene NPs. They observed that, depending on the specific ratio between the peptide and particle concentration, the NP effects can vary from acceleration of the fibrillation process to inhibition (Figure 1).

## 8. Amyloids, Plastics, and the Environment

Further evidence of a possible role of MNPs in protein folding aberrations comes from findings obtained in laboratory settings and in the environment.

Prions and amyloids interact with the plastic surface of disposable laboratory tools such as centrifuge tubes, and this interaction influences amyloid fibrillation. A study tested the propensity of amyloids to bind the surface of centrifuge tubes made of different materials: (i) polycarbonate; (ii) a copolymer of polystyrene and acrylonitrile; (iii) polystyrene; and (iv) polypropylene. Polystyrene tubes were the ones that showed more amyloid absorbance [83]. This evidence is supported by a second study that showed how amyloid β fibrils adsorb to the plastic surface of cell culture plates and centrifuge tubes. Interestingly, the authors have found that the adsorption is enhanced by the presence of complex biological samples. This may suggest that, in real-life conditions, biological fluids and components could affect amyloid and plastic interactions, a finding that is in line with studies on the effects of the protein corona on amyloids [84,85]. This interaction is not limited to fully formed amyloid fibers. Self-propagating amyloid isoforms of the vitro-formed recombinant prion protein (rPRP) are adsorbed to the surface of the plastic tube during the amyloid conversion process [86].

This interaction of amyloids and plastics was further confirmed in a study where the authors used amyloids to precipitate dispersed and negatively charged MPs of 500 nm from aqueous solutions. The fibers used where obtained from lysozyme, but the authors argue that finding can be generalized to other proteins, although specific modifications may be required [87].

The interaction has also been exploited for the development of bioplastics. Thanks to their biophysical properties and mechanical and chemical stabilities, amyloid fibrils have been used as building blocks in several applications, such as emulsions, membranes, and gels with high performances. When mixed with a plasticizer and a water-soluble polymer, the amyloids, through a fibrillization process, organized themselves in fibers and constituted a suitable building block for a new class of hybrid bioplastics [88]. Moreover, amyloid lysozyme fibrils were also conjugated with polyethyleneimine to create a new tool for the removal of lead (II) from water [89].

All this can be achieved also because amyloids and plastics share a similar polymerization process that can be mediated by nucleating agents [90]. Interestingly, fibrillation can vary by the use of different plastics [91]. Overall, these studies support a connection between protein folding, amyloids, and plastics.

## 9. MNPs and Amyloidosis

On one hand, exposure to MNPs has been linked to various human conditions. One of the best-characterized examples is the association between vinyl chloride and tumor risk. Vinyl chloride is a component of PVC, exposure to which is thought to generate hepatocellular carcinoma-inducing mutations, particularly mutations in oncogene K-ras-2 [92]. On the other hand, the potential role of MNPs in disorders characterized by aberrant protein folding and amyloids has been largely overlooked.

Neurodegenerative diseases such as AD and PD are not fully understood, though nearly every one of them is thought to originate from the aggregation of misfolded proteins. AD is the most common cause of dementia and the sixth leading cause of death in America, responsible for more than 120,000 lives lost annually [93]. Although AD is generally thought to occur sporadically or due to genetic factors, it has also been associated with numerous environmental and behavioral factors [94]. A recent in vitro study using zebrafish found that diesel exhaust disrupts autophagy in neurons, leading to cell death and altered behavior; this study offers a mechanism that may underlie the link between air pollution and neurodegenerative diseases [95]. With evidence showing that NPs can cross the blood–brain barrier, interact with intracellular amino acids, and distort native protein folding, it is plausible that they can trigger the aggregation of amyloid proteins, thereby effectively creating the precursors for AD. The theory that AD can be triggered by environmental causes is supported by the relatively low incidence of genetic cases of AD and could explain, at least in part, the sporadic forms of this neurodegeneration [96]. Interestingly, new theories suggest that central neurodegeneration starts in the periphery, namely, in enteric neurons, the cells forming the complex network responsible for the regulation of the functions of the gastrointestinal tract. In recent decades, an increasing number of studies have indicated that the enteric neuron system, possibly through interplay with the gut microbiome, plays an important role in the manifestation of various neurodegenerative disorders, such as AD, PD, and prion diseases [97,98,99,100,101]. In these pathologies, enteric neurons not only present similar pathological phenotypes as central neurons but are affected at even earlier stages of disease compared to central neurons [102]. Enteric neurons, due to their location in the gut wall, are likely the first neurons exposed to MNPs, and the effects of NPs on their morphology and functionality have been hypothesized (Ref. [103] and personal observation of the authors), but not yet extensively studied. However, consistent findings already suggest that the functionality of the digestive system may be altered by MNPs [104,105,106]. Thus, our involuntary consumption of plastic pollution may be a critical factor triggering the initial development of neurodegenerative diseases or influencing the speed of illness progression.

While many amyloidosis occur exclusively in the central nervous system [107], aggregates can form and deposit either systemically or in single organs [108]. Since MNPs can target different organs, as has been demonstrated in numerous model organisms, they may also trigger other, organ-specific or systemic proteinopathies. Systemic amyloidosis is a rare multisystemic condition that can affect the heart, kidneys, nerves, liver, lungs, and bowel. It is caused by at least 14 different proteins that can form extracellular protein fibrils deposition in these tissues, causing toxicity and eventually death [109]. The two most common systemic amyloidosis are immunoglobulin light chain (AL) amyloidosis and amyloid TTR (ATTR) amyloidosis. AL amyloidosis often arises from a plasma cell dyscrasia, wherein the monoclonal immunoglobulins are the precursor amyloid protein [110]. Misfolded monoclonal light chains deposit in various organs, though mainly in the heart, leading to cardiac pathologies [108]. Alternatively, mutant proteins, such as transthyretin and β2 microglobulin, can target individual organs, commonly the heart, liver, and kidneys, leading to impairment of organ function and metabolic diseases such as heart failure, and liver disease, renal dysfunction, and diabetes [111,112].

ATTR amyloidosis is caused by mutations in the transporter of thyroxine and retinol-binding (TTR) protein. In physiological conditions, the protein forms a stable tetramer. Pathogenic mutations affect the tetramer formation leading to the polymerization of the monomeric protein into toxic amyloid fibrils [113].

To our knowledge, no studies have investigated a possible link between systemic amyloidosis and MNPs exposure.

## 10. Conclusions

Pollution due to plastic is an insidious world health issue increasing every second. The damage extends to nearly every living organism, and further research is needed to better characterize the biological and clinical effects of plastics consumption. Currently, the literature suggests that MNPs are prone to enter the cell membranes and interact with intracellular proteins, possibly disrupting their native structures and thereby altering their functionality. This process may trigger or accelerate the development of many neurodegenerative diseases. However, the mechanisms and pathways that may be involved are still far from being understood, and also, the putative impact on human health needs to be shown [114]. Currently, all studies have been carried out either in vitro or in animal models. Future research should therefore also involve the study of cellular mechanisms of MNP internalization, the interaction with different subcellular structures and specific proteins, and the relationship between their chemical characteristics and protein misfolding in the context of human health. Specifically, research should focus on: (i) the characterization of MNPs in the environment; (ii) their ability to interact with body fluids after uptake by both animal models and humans (for instance, by characterization of eventual formation of protein coronas); (iii) the exploration of experimental or chemical methods to mimic the creation of MNPs with similar characteristics to the ones found in the environment (for carefully controlled laboratory studies); (iv) the study of the mechanisms of MNP uptake and elimination by different cell types (e.g., enterocytes, blood cells, or neurons); (v) the measurement of MNP cell toxicity, with a focus on neurons; (vi) the ability of MNP to cross the blood–brain barrier; (vii) the biophysical study of the influence of different MNPs on protein folding and misfolding; (viii) the analysis of postmortem brain tissues of AD and other neurological patients to detect the presence of any MNP compared to healthy controls; (ix) the analysis of tissue biopsies obtained from patients suffering from amyloidosis; and (x) the study of the effects of MNPs on the progression of amyloidosis (both systemic or causing neurodegeneration) in suitable animal models.

All this will allow a comprehensive understanding of whether MNPs have a role in the pathogenesis of amyloidosis. 

## Data Availability

Not applicable.
